# Effect of Genetic and Laboratory Findings on Clinical Course of Antisynthetase Syndrome in a Hungarian Cohort

**DOI:** 10.1155/2018/6416378

**Published:** 2018-10-25

**Authors:** Katalin Szabó, Levente Bodoki, Melinda Nagy-Vincze, Anett Vincze, Erika Zilahi, Peter Szodoray, Katalin Dankó, Zoltán Griger

**Affiliations:** ^1^University of Debrecen, Faculty of Medicine, Division of Clinical Immunology, Móricz Zs. krt. 22, 4032 Debrecen, Hungary; ^2^University of Debrecen, Faculty of Medicine, Department of Laboratory Medicine, Nagyerdei krt. 98, 4032 Debrecen, Hungary; ^3^Institute of Immunology, Rikshospitalet, Oslo University Hospital, Sognsvannsveien 20, 0372 Oslo, Norway

## Abstract

The aim of this study was to determine the clinical, serological, and genetic features of anti-Jo-1 positive antisynthetase patients followed by a Hungarian single centre to identify prognostic markers, which can predict disease phenotypes and disease progression. It was a retrospective study using clinical database of 49 anti-Jo-1 positive patients. 100% of patients exhibited myositis, 73% interstitial lung disease, 88% arthritis, 65% Raynaud's phenomenon, 43% fever, 33% mechanic's hand, and 12% dysphagia. We could detect significant correlation between anti-Jo-1 titer and the CK and CRP levels at disease onset and during disease course. HLA DRB1⁎03 positivity was present in 68.96% of patients, where the CK level at diagnosis was significantly lower compared to the HLA DRB1⁎03 negative patients. HLA DQA1⁎0501-DQB1⁎0201 haplotype was found in 58.62% of patients, but no significant correlation was found regarding any clinical or laboratory features. Higher CRP, ESR level, RF positivity, and the presence of fever or vasculitic skin lesions at the time of diagnosis indicated a higher steroid demand and the administration of higher number of immunosuppressants during the follow-up within anti-Jo-1 positive patients. The organ involvement of the disease was not different in HLA-DRB1⁎0301 positive or negative patients who were positive to the anti-Jo-1 antibody; however, initial CK level was lower in HLA-DRB1⁎0301 positive patients. Distinct laboratory and clinical parameters at diagnosis could be considered as prognostic markers.

## 1. Introduction

Idiopathic inflammatory myopathies (IIMs) are systemic autoimmune connective tissue diseases characterized by chronic muscle inflammation resulting in progressive symmetrical muscle weakness with elevated serum levels of muscle enzymes, electromyographic abnormalities, and characteristic mononuclear inflammatory infiltrates in muscle biopsy specimens. Inflammation of skeletal muscles and internal organs underpins IIM, leading to irreversible damage and even death. The most commonly used criteria for the clinical diagnosis of IIMs were proposed by Bohan and Peter in 1975 [1]. Autoantibodies are of great importance for the diagnosis of many systemic autoimmune rheumatic diseases, including IIMs. Myositis-associated autoantibodies (MAAs) are those that appear in myositis overlap syndromes and in other connective tissue diseases, which correlate with certain clinical and/or pathophysiological conditions of myositis [2–8]. The myositis-specific autoantibodies (MSAs) are useful markers for clinical diagnosis, classification, and prediction of the prognosis of the IIM. Approximately 50-70 % of IIM patients have MSAs in their sera [9, 10]. The most frequent MSA in the serum of patients with myositis is anti-Jo-1 [11]. Presence of anti-Jo-1 defines a distinct clinical phenotype, antisynthetase syndrome (ASS), which is characterized by poor prognosis, and multiple organ involvement, such as myositis, interstitial lung disease (ILD), arthritis, Raynaud's phenomenon, mechanic's hand, skin rashes, and fever [12]. Recently, new classification criteria for IIM were developed [13], where the presence of anti-Jo-1 antibody plays an important role in the scoring system with the highest score point.

Autoimmune processes observed in inflammatory myopathies are not fully understood, but it seems that genetic and environmental factors (viral infections, UV light) are likely to interact to confer risk for developing chronic inflammatory diseases such as polymyositis (PM) and dermatomyositis (DM). It is known that the pathogenesis of IIMs involves strong interactions between dendritic cells, activated Th1 and Th17 cells, B cells, muscle cells, genes, and environmental factors [14]. The autoimmune origin is supported by derailed cellular and humoral immune processes [15]. Ethnic differences and the HLA-associations suggest that genetic factors may play a part in the pathomechanism [16].

Considering the etiology of patients with anti-Jo-1 antibody, the following are of great importance. Some special genes may play a role in the development of ASS antibodies. Human leukocyte antigen genes on chromosome 6, particularly HLA-DRB1*∗*0301 and the linked allele DQA1*∗*0501, have the strongest associations with the presence of anti-Jo-1 antibody in Caucasian patients [17]. HLA-DQA1*∗*0501 and HLA-DQA1*∗*0401 are associated with this antibody in African-Americans and Hispanics. Smoking appears to be associated with an increased risk of having anti-Jo-1 in HLA-DRB1*∗*03-positive IIM cases. Chinoy et al. [18] hypothesized that the interaction between HLA-DRB1*∗*03 and smoking may lead to the development of anti-Jo-1 antibodies.

The aims of this study were (1) to determine the demographic, clinical, serological, laboratory, and genetic features of Hungarian anti-Jo-1 positive myositis patients; (2) to find any significant correlation between having the HLA-DRB1*∗*0301 allele and the presence of distinct organ involvement; (3) to assess relevant markers, or clinical features at the onset of the disease, which can predict the progression of myositis, or the response to the therapy.

## 2. Materials and Methods

### 2.1. Patients

Data of 49 anti-Jo-1 positive myositis patients were analyzed retrospectively. All patients are followed by the Department of Clinical Immunology at the University of Debrecen, Hungary, and medical files of the patients were reviewed. The median follow-up was 10.1 ± 6,51 years. This study meets and is in compliance with all ethical standards of medicine. Informed consent was obtained from all of the subjects. This study is in compliance with the Declaration of Helsinki. Diagnosis was made in each case according to the Bohan and Peter criteria and all patients had a definitive or probable diagnosis of idiopathic inflammatory myopathy. None of these patients had other connective tissue disorders or myopathy; secondary Sjögren's syndrome was excluded. ILD involvement was investigated initially by pulmonary function test and high-resolution computed tomography (HRCT). Prognosis was assessed by determination of mortality during the follow-up. In addition higher maintenance doses of steroids and a need of a higher number of immunosuppressants (including cyclophosphamide) have been used as surrogated markers of bad prognosis. The occurrence of vasculitic skin lesions (small vessel vasculitis or capillaritis) was assessed clinically based on the presence of purpura and/or skin ulcers with or without histology.

### 2.2. Immunoserology

The presence of anti-Jo-1 antibody was detected by the Regional Immunological Laboratory in Debrecen using immunoblot [ORGENTEC; ORG 760; ORGENTEC Diagnostika GmbH, Germany], with subsequent confirmation by enzyme linked immunosorbent assay (ELISA). Titers of the antibodies against extractable nuclear antigen (ENA) complex, anti-SS-A (Ro) and anti-Jo-1 antibodies, were measured (HYCOR Biomedical Inc., CA, USA) using this latter method. It was assessed at the beginning of the disease, and the titer of anti-Jo-1 was followed during disease progress.

### 2.3. Genotyping

High molecular weight DNA for genotyping was extracted from peripheral blood, which was collected in EDTA Vacutainers. Genomic DNA was extracted according to the manufacturer's recommendation using a QIAamp DNA Blood Mini Kit (QIAGEN GmbH, Germany). DNA was quantified by ultraviolet absorption at 260 and 280 nm and stored at -20°C until analyzed. Human leukocyte antigen (HLA)-DRB1, -DQA1, and -DQB1 genotyping was performed with sequence-specific primers (Olerup SSP, GenoVision, Oslo, Norway). All samples were processed according to the manufacturer's instructions based on polymerase chain reactions (PCR). HLA genotypes were determined on the basis of the PCR product pattern obtained using 2% agarose gel electrophoresis.

### 2.4. Statistical Analysis

All data were evaluated using SPSS 20.0 statistical software and adequate statistical probes (Pearson Chi-square (*χ*2), Fisher's exact test, Spearman's correlation). A p value less than 0.05 was regarded as statistically significant.

## 3. Results

### 3.1. Demographic Data and Genetic Investigation

The data of 49 myositis patients with anti-Jo-1 antibodies were evaluated.  Table 1 shows demographic clinical and genetic characteristics of the patients. Considering the symptoms of the classical ASS, the frequency of the features was as follows: myositis 100%; ILD 73%; arthritis 88%; Raynaud's phenomenon (RP) 65%; fever 43%; and mechanic's hand 33%. Other skin symptoms were much rarer than mechanic's hand. HLA-DRB1, -DQA1, and -DQB1 genotypes of 29 patients with anti-Jo-1 positivity were determined using commercial sequence-specific oligonucleotide kit. HLA-DR3 (HLA-DRB1*∗*03) alleles were present in 20 (68.96%) anti-Jo-1 positive patients. HLA-DQA1*∗*051-DQB1*∗*0201 haplotype was represented in 17 (58.62%) patients. The correlation of HLA-DRB1*∗*03 positivity and different parameters of myositis (organ involvement, laboratory parameters, serological status) was also investigated. We found that HLA-DRB1*∗*03 positivity was associated with lower initial CK level in patients with anti-Jo-1 positivity; however no other clinical parameters were influenced by the presence or absence of HLA-DRB1*∗*03 genotype (Table 1). In addition, serological or therapeutic features of the patients were also not affected by the HLA-DRB1*∗*03 genotype (data not shown). Five out of the 49 patients died during the 10.1 years median follow-up time. None of the investigated parameters were significantly associated with the mortality (data not shown).

### 3.2. Autoantibodies

**Figure 1:**
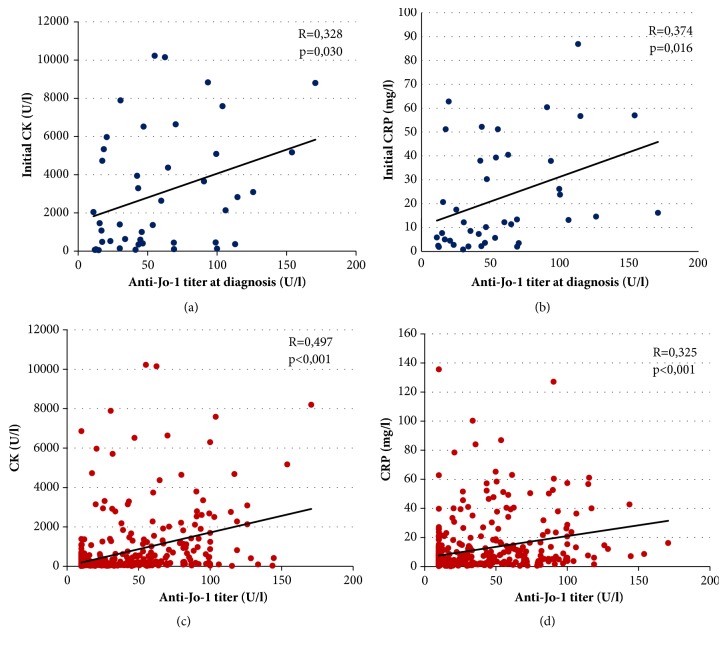
Correlation between anti-Jo-1 titer and CK/CRP levels at diagnosis and during disease progress. (a) Correlation between the initial anti-Jo-1 titer and the first CK level. (b) Correlation between the initial anti-Jo-1 titer and the first CRP level. (c) Correlation between anti-Jo-1 levels during disease course to the corresponding CK level. (d) Correlation between anti-Jo-1 levels during disease course to the corresponding CRP level. (CK: creatine kinase; CRP: C-reactive protein.)

Multiple laboratory parameters were followed during disease progress. The average creatine kinase (CK) level at diagnosis was 3003.25 U/L and lactate dehydrogenase (LDH) level 922.33 U/L, whereas the average C-reactive protein (CRP) was 22.49 mg/L and erythrocyte sedimentation rate (ESR) 24.24 mm/h. The anti-Jo-1 titer at diagnosis showed significant correlation with both the initial CK (p=0.03; R=0.328) and CRP levels (p=0.016; R=0.374). In addition, both CK levels (p<0.001) and CRP levels (p<0.001) showed significant positive correlation with the anti-Jo-1 titer collected at the same time during disease course (Figures 1(a)–1(d)).

The presence and clinical significance of other autoantibodies were also investigated. The most frequently found antibody in the sera of anti-Jo-1 positive patients was anti-SSA (17/49; 35 %). We compared the clinical and laboratory findings of the anti-SSA positive group with anti-SSA negative patients (Table 2). We found that the age at disease onset and the frequency of interstitial lung disease in patients with anti-SSA were significantly lower (p=0.004 and p=0.039). In contrast, the minimal stable dose of steroid was significantly higher in the SSA positive group (p=0.031). It should be emphasized that skin erosions were present only in patients having anti-SSA antibodies (p=0.037).

### 3.3. Therapy

Almost all available pharmacologic therapies were used during the disease course of the investigated patients and all of the patients were instructed to do exercises regularly. Concerning medications, 10 patients (20.41%) received only methylprednisolone therapy, whereas two patients (4.08%) refused the steroid treatment. 26 (53.06%) patients got steroid and immunosuppressive drug therapy, more than half of them at least two different drugs. The frequency of the used immunosuppressant was the following: methotrexate (MTX: 21 patients), cyclophosphamide (Cyc: 20 patients), azathioprine (AZA: 17 patients), cyclosporine (CSA: 13 patients), hydroxychloroquine (HQ: 7 patients), and sulfasalazine (6 patients). During the disease course nine (18.37%) patients received intravenous immunoglobulin (IVIG) treatment, one patient rituximab alone, and one patient IVIG with rituximab.

We compared the clinical symptoms and laboratory parameters found at the diagnosis in those patients who received Cyc with the group that did not during the disease course. In the “cyclophosphamide group” regarding the clinical parameters, the frequency of interstitial lung disease (90% versus 60%, p=0.024), fever (60% versus 31%, p=0.044), Raynaud's phenomenon (85% versus 51%, p=0.016), and vasculitis-like skin lesion (25% versus 3.4%, p=0.035) was significantly higher at the disease onset. Focusing on laboratory parameters, the CRP level was also significantly higher in this subgroup (31.18±21.77 versus 16.921±20.85, p=0.042) at diagnosis.

In addition, we compared the clinical symptoms and laboratory parameters at diagnosis of those patients who received only one immunosuppressant with those who were treated with more. The second group had more frequent fever (68% versus 22%, p=0.001), vasculitis-like skin lesions (27% versus 0%, p=0.005), and higher CRP level at disease onset (30.461±21.51 versus 16.25±20.9, p=0.039). Rheumatoid factor (RF) was detectable more frequently in the second group (59% versus 18%, p=0.003).

Finally, based on the minimum stable dose of methylprednisolone treatment, the patients were categorized into the following two groups: (i) by 65% (n=32) of all patients, less than 8 mg; (ii) 35 % (n=15) of the patients more than 8 mg to control disease activity. We could detect that CRP (17.84±18.32 versus 36.34±25.39; p: 0.014); ESR (19.81±10.4 versus 33.87±22.11; p: 0.032); and the presence of fever (34% versus 67%; p: 0.038) at diagnosis were significantly higher in the group receiving more than 8 mg methylprednisolone during disease course (Table 3).

## 4. Discussion

We can summarize our recent work as follows: (1) the phenotype of the disease is not different in HLA-DRB1*∗*0301 positive or negative patients who has the anti-Jo-1 antibody, but the initial CK level was significantly higher in the HLA-DRB1*∗*0301 negative patients; (2) distinct laboratory parameters measured at disease onset (high CRP, high ESR, anti-SSA, RF) and the presence of certain clinical symptoms (fever, vasculitis-like skin lesions) refer to a more difficult disease course, requiring higher steroid maintenance dose and multiple immunosuppressant treatments during the follow-up of patients with ASS; and (3) anti-Jo-1 titer and the CK and CRP levels were positively correlated at disease onset and during disease course.

We published earlier the phenotypes and organ involvements of anti-Jo-1 positive patients in Hungarian [19]. In this study we have compared the phenotypes and laboratory data of the patients with newly determined different genotypes. Based on our genetic data it seems that having anti-Jo-1 antibodies will determine the phenotypes of the patients more than the presence or absence of certain haplotypes. The comparison of the HLA-DRB1*∗*03 positive and negative patients with anti-Jo-1 antibody showed that none of the clinical symptoms, organ involvements, minimal stable steroid dose, or inflammatory parameters at disease onset differed in the two groups. Thus, it seems that HLA-DRB1*∗*03 positivity is one of the major factors which can play a role in the development of anti-Jo-1 antibody, but the organ involvement, i.e., the main phenotype of the disease, is not different in patients who has the antibody and the disease symptoms. Interestingly, the initiating CK level was higher in the HLA-DRB1*∗*03 negative group, which may indicate a more severe myositis; however disease severity, including myositis, is affected by certain other parameters (muscle force, gastrointestinal involvement, severity and extent of extramuscular involvements, etc.). Therefore further work and investigation of a larger patient population are required including the assessment of disease activity core set measures [20–22] to determine the impact of this phenomenon.

Another aim of this study was to find potential markers, or clinical features at the onset of the disease, which can predict the response to the therapy. This could be notably important, since before the validation of assessment and improvement criteria, there were no controlled drug studies for the treatment because of the low incidence and the heterogeneity of myositis subtypes. Therefore, recommendations are mainly based on clinical observations. The early administration of immunosuppressants (MTX, AZA, Cyc, CSA) is considered first-line adjuncts to glucocorticoid therapy, or as steroid sparing agents, although there is limited evidence for its use [23]. With no clearly superior agent, the choice of immunosuppressive agent remains dependent on patient factors and clinician preference. Cyclophosphamide can increase the vital capacity and the diffusion capacity and decrease the extent of alveolitis; in addition it also improves the muscle strength and function [24]. Cyclosporine is also effective and substantially safe in patients with anti-Jo-1 ASS with corticosteroid-refractory ILD [25]. According to Marie et al. [26] in anti-Jo-1 patients with severe oesophageal manifestations, combined high-dose steroids and IVIGs might be proposed as the first-line therapy. The Rituximab in Myositis (RIM) trial showed that there were no significant differences in the 2 treatment arms for the primary and secondary end points, but 83% of adult and juvenile myositis patients with refractory disease met the DOI [27]. Moreover subanalysis revealed that antisynthetase patients responded better and other limited but encouraging results showed that rituximab may stop the progression of ASS-associated ILD [28, 29].

Our second novel finding was that in those patients who needed at least two different immunosuppressants, the occurrence of fever was significantly higher at disease onset and their CRP levels were also significantly higher. Similarly, the presence of fever and high CRP at diagnosis was found more often in those patients, who were treated later with cyclophosphamide. The presence of RF and the vasculitis-like skin lesions were also more frequent in these patients. The mortality results of our cohort did not show any significant associations, which could be accountable for the low number of patients enrolled. Nevertheless, our results indicate that higher steroid doses were used in those patients, whose ESR and CRP levels were high at disease onset or in whom fever was present (Table 3). This suggests that these factors could be used as possible prognostic markers at disease onset. In these cases the physician could predict a more difficult disease course with higher steroid demand and a need of a higher number of immunosuppressants, which might be surrogate markers of poor prognosis and treatment failure. Therefore further longitudinal, ideally prospective studies are required to assess the exact predictive value of the abovementioned parameters on the prognosis and treatment response using the newly developed therapeutic response criteria [30].

The comparison of our data with results of other workgroups [26, 31–38] is challenging, since ASS is a complex disease and the clinical picture might evolve during the follow-up and there are no well-established classification criteria. Some of the groups were selecting ASS patients with a dominant muscle disease, but in other cohorts [35, 37] the selection was made based on the positivity of anti-Jo-1 test and the presence of at least one clinical finding between arthritis, myositis, and ILD. That could be the reason of marked differences in the presence of myositis within the different ASS population. In our cohort only those patients who fulfilled the probable or definite Bohan and Peter criteria, were included, and that is why the presence of myositis was 100 %, and anti-Jo-1 positive patients without myositis were excluded. Therefore the comparison of the phenotypes of the patients is defying. However, it seems that the majority of our demographic (average age at disease onset) and some of the clinical data of our patients were similar to those found in the literature. The causes of the differences may be the distinct genetic and environmental factors or the selection criteria of the cohorts discussed above. Therefore the development of classification criteria for ASS based on differential impact for various clinical, pathological, and serological variables is needed, and this was proposed recently by others as well [39].

Intensive research is under way to show that the titer of anti-Jo-1 autoantibody is also part of the immune process and appears not just as a marker. Some publications have highlighted that anti-Jo-1 autoantibodies may play a role in disease propagation and pathogenesis. Eloranta et al. [40] concluded that immune complexes containing either anti-Jo-1 or anti-Ro in the presence of ribonucleic acid (RNA) may act as endogenous inducers of type 1 interferon-*α*. Howard et al. [41] investigated the chemoattractant properties of several aminoacyl-tRNA synthetase molecules and demonstrated that histidyl (Jo-1) aminoacyl-tRNA synthetase can induce leukocyte migration. The authors suggested that autoantigenic aminoacyl-tRNA synthetases are overexpressed in damaged muscle cells and their proinflammatory properties promote the immune response, which leads to the development of myositis. The pathogenic role of anti-Jo-1 antibody is supported by evidence that anti-Jo-1 levels showed modest correlation with CK, myositis, and joint disease activity [31]. We found in our population that there was a significant correlation between the initial CK and CRP levels and the anti-Jo-1 titer at diagnosis. Strong associations of anti-Jo-1 titer and CK/CRP levels during disease progress were also detected. These data argue for the assumption that the presence of ant-Jo-1 titer is not only a diagnostic marker. Therefore we propose that measurement of the anti-Jo-1 titer might be an advantageous additional component of standard disease activity core set measures [20] during disease course in the antisynthetase patients.

Anti-SSA antibody is often found in the sera of anti-Jo-1 positive myositis patients [21]. The cooccurrence of anti-SSA/Ro may have an effect on ASS prognosis: it was associated with more severe ILD [42, 43], which was not confirmed in a larger cohort. Marie et al. failed to show a significant difference regarding progression of ILD between patients with and without anti-Ro52 antibody and concluded the association of more severe myositis, joint impairment, and increased risk of cancer with the coexistence of anti-Ro52 antibody in anti-Jo-1 positive patients [22]. We could notice that the anti-SSA positive patients were younger, the presence of skin erosions was more extensive, and ILD was less frequent compared to the SSA negative group [Table 2]. In our population the steroid demand was higher in the SSA positive group, which could be a surrogate marker of a more progressive disease; however the prevalence of ILD was lower in this group. The cause of this phenomenon needs further clarification; thus we did not investigate the severity of lung involvement. Nevertheless it seems that having anti-SSA antibody may define a distinct subgroup within the ASS patients and further studies are required to better characterize the phenotype of ASS associated with anti-SSA and to improve treatment for these patients.

## 5. Conclusions

We conclude that the organ involvement of anti-Jo-1 positive patients with myositis was not affected, however initial CK level was influenced by the HLA-DRB1*∗*03 genotype. There is a positive correlation between anti-Jo-1 titer and the CK and CRP levels at disease onset and during disease course. Distinct laboratory results measured at the diagnosis (higher CRP, -ESR level; -RF positivity) and the presence of certain clinical symptoms (fever, vasculitic skin lesions) may indicate a higher steroid demand and more difficult disease course within anti-Jo-1 positive antisynthetase patients. We believe that our findings may have potential support during the care of ASS patients, but further investigations are required to assess the exact impact of these factors on prognosis and treatment response.

## Figures and Tables

**Table 1:** Demographic, clinical, and genetic results of anti-Jo-1 positive patients.

	**Anti-Jo-1 positive patients**		**HLA-DRB1** **∗** **03 positive patients**		**HLA-DRB1** **∗** **03 negative patients**		**p value**
Number of patients:	49		20		9		-
Male/Female:	7/42		1/19		2/7		0.22
Average age at disease onset ± SD (youngest-oldest):	43.4 ±13.28 (18-70)		40.4±13.9		40.22±14.55		0.703
Median follow-up time ± SD (years)	10.1 ±6.51		11.5±6.79		12.33±5.34		0.748

Average CK at diagnosis (U/l) ± SD	3003.25 ±3101.8		2816.30 ±2417.36		5969.44 ±3842.89		**0.045**
Average LDH at diagnosis (U/l) ± SD	922.33 ±635.32		891.78±661.3		1292.57 ±737.948		0.2
Average CRP at diagnosis (mg/l) ± SD	22.49 ±22.09		18.32±22.55		23.99±17.17		0.542
Average ESR at diagnosis (mm/h) ± SD	24.24 ±15.96		21.50±13.57		24.89±14.46		0.547

myositis:	49/49	100%	20/20	100%	9/9	100%	1.0
ILD/alveolitis:	35/48	73%	15/20	75%	7/9	78%	1.0
arthritis/arthralgia:	43/49	88%	16/20	80%	8/9	89%	1.0
dysphagia:	6/49	12%	3/20	15%	1/9	11%	1.0
fever:	21/49	43%	7/20	35%	6/9	67%	0.226
Raynaud's phenomenon:	32/49	65%	10/20	50%	8/9	89%	0.096
mechanic's hand:	16/49	33%	5/20	25%	5/9	56%	0.205
subcutaneous calcinosis:	3/49	6%	1/20	5%	1/9	11%	0.532
SSA positivity:	17/49	35%	7/20	35%	2/9	22%	0.675
mortality:	5/49	10%	3/20	15%	0/9	0%	0.532
Maintenance dose of steroid (mg):	5.57±8.44		3.90±3.81		3.44±3.644		0.765

**Table 2:** Detailed comparison of anti-Jo-1+/anti-SSA+ and anti-Jo-1+/anti-SSA-patients.

	**SSA positive patients**		**SSA negative patients**		**p**
**Demographic data **					

Number of patients:	17		32		-
Male/female:	1/16		6/26		0.397
*Average age at disease onset ± SD (youngest-oldest):*	*36.12±11.08* *(21-58)*		*47.22±12.87* *(18-70)*		*0.004*

**Laboratory parameters**					

Average CK at diagnosis (U/l) ± SD:	3484.87±3549.27		2754.14±2878.91		0.465
Average LDH at diagnosis (U/l) ± SD:	871.33±491.29		949.64±707.46		0.705
Average CRP at diagnosis (mg/l) ± SD:	24.76±22.21		21.18±22.35		0.623
Average ESR at diagnosis (mm/h) ± SD:	26.94±19.34		22.81±13.98		0.394

**Clinical symptoms**					

Myositis:	17	100%	32	100%	1
*Interstitial lung disease:*	*9*	*53%*	*26*	*81%*	*0.039*
Arthritis:	15	88%	28	88%	1
Raynaud's phenomenon:	13	76%	19	59%	0.231
Dysphagia:	2	12%	4	13%	1
Fever:	10	59%	11	34%	1

Skin lesions (all):	9	53%	18	56%	1

there from:					
Mechanic's hand:	6	35%	10	31%	0.774
Gottron-papule:	2	12%	4	13%	1
Gottron-sign:	2	12%	4	13%	1
Scarf –sign:	1	6%	1	3%	1
V-sign:	2	12%	2	6%	0.607
Heliotrope rash:	0	0	3	9%	0.542
Periorbital oedema:	1	6%	2	6%	1
Alopecia:	1	6%	2	6%	1
Vasculitis-like skin lesion:	3	18%	3	9%	0.405
Livedo reticularis:	3	18%	1	3%	0.114
*Skin erosion:*	*3*	*18%*	*0*	*0*	*0.037*
Teleangiectasia:	1	6%	5	16%	0.65
Subcutaneous calcinosis:	1	6%	2	6%	1

**Therapy**					

**Maintenance dose of steroid (mg) ± SD:**	**9.53±12.56**		**3.47±3.95**		**0.031**

SSA: Sjögren's-syndrome-related antigen A; SD: standard deviation; CK: creatine kinase; LDH: lactate dehydrogenase; CRP: C-reactive protein; ESR: erythrocyte sedimentation rate.

**Table 3:** Detailed comparison according to the stable dose of methylprednisolone.

**Minimal stable steroid dose: (n=47)**	**5.57mg±8.436**				
	**<8mg**		**≥8mg**		**p**
Number of patients:	32		15		-
Male/Female:	6/26		1/14		0.404
Mean age at disease onset ± SD (youngest-oldest):	43.91±13.18(18-70)		41.47±14.29(19-67)		-

**Clinical symptoms and laboratory parameters:**					

myositis:	32	100%	15	100%	1
LDH at diagnosis(U/l)±SD:	1035.26±707.38		775.93±463.93		0.224
CK at diagnosis(U/l)±SD:	3447.64±3176.22		2531.86±2982.12		0.374
ILD/alveolitis:	24	75%	11	73%	1
arthritis:	29	91%	12	80%	0.367
*CRP at diagnosis(mg/l)±SD: *	*17.84±18.32*		*36.34±25.39*		*0.014*
*ESR at diagnosis(mm/h)±SD:*	*19.81±10.4*		*33.87±22.11*		*0.032*
Raynaud-phenomenon:	21	66%	10	67%	0.944
dysphagia:	2	6%	4	27%	0.072
*fever:*	*11*	*34%*	*10*	*67%*	*0.038*
Skin lesion:	15	47%	11	73%	0.089

SD: standard deviation; LDH: lactate dehydrogenase; CK: creatine kinase; ILD: interstitial lung disease; CRP: C-reactive protein; ESR: erythrocyte sedimentation rate.

## Data Availability

All patients are followed by the Department of Clinical Immunology at the University of Debrecen, Hungary. Medical files of the patients (available only for our myositis workgroup because of ethical reason) were reviewed by the help of MedSol Database.
